# Slaughter of pregnant cattle in German abattoirs – current situation and prevalence: a cross-sectional study

**DOI:** 10.1186/s12917-016-0719-3

**Published:** 2016-06-07

**Authors:** Patric Maurer, Ernst Lücker, Katharina Riehn

**Affiliations:** Institute of Food Hygiene, Centre of Veterinary Public Health, Faculty of Veterinary Medicine, University of Leipzig, An den Tierkliniken 1, 04103 Leipzig, Germany; Faculty of Life Sciences, Hamburg University of Applied Sciences, Ulmenliet 20, 21033 Hamburg, Germany

**Keywords:** Veterinary public health, Animal welfare, Pregnancy, Gravid cattle, Foetus, Abattoir, Slaughterhouse, Consumer protection

## Abstract

**Background:**

The slaughter of pregnant cattle and the fate of the foetuses are relatively new subjects in the field of animal welfare. The Scientific Committee on Veterinary Measures relating to Public Health (SCVPH), however, does not believe this topic to be a critical issue because of the hitherto supposed rare occurrence of this practice. Some previous studies though, contradict this assessment, emphasising its relevance to animal welfare. With regard to the heterogeneous study design of previous investigations, the objective of this study is to evaluate the current situation concerning the slaughter of pregnant cattle in different German abattoirs. Additionally, the prevalence was assessed semi-quantitatively on the basis of a cross-sectional, voluntary and anonymous survey that was conducted amongst senior veterinary students of the University of Leipzig from 2010 until 2013.

**Results:**

Of 255 evaluable questionnaires, 157 (63.6 %) mention the slaughter of pregnant cattle, corresponding to 76.9 % of all visited abattoirs. Slaughter of pregnant cattle is reported often (>10 % of females) in 6 (3.8 %), frequently (1–10 % of females) in 56 (35.7 %), and rarely (<1 % of females) in 95 (60.5 %) of all cases (*n* = 157) respectively. About 50 % of these animals were reported to be in the second or third stage of gestation. 15 (10.6 %) of 142 questionnaires providing information about the foetus, state that the foetus showed visible vital signs after the death of the mother, but in one case the foetus was euthanized subsequently.

**Conclusions:**

The results show that the slaughter of pregnant cattle is a common and widespread practice in German abattoirs. The SCVPH’s assumption that pregnant cattle are only slaughtered in rare exceptional cases can no longer be maintained. The high proportion of foetuses in the second and third gestational stage must also be considered. In this context the implementation of suitable studies and detailed analysis of the current situation is indispensable to ensure the high standards in animal welfare in Germany and Europe.

## Background

### Current developments in public opinion and politics

Germany has one of the strictest animal welfare laws worldwide. There is no other country in the European Union (EU), which integrated animal welfare into its constitution – in Germany animal welfare was made a national objective already in 2002 [1].

In recent years the slaughter of pregnant cattle has gained importance in the public debate on animal welfare. Due to scientific work by, amongst others, Peisker et al., Riehn et al., Di Nicolo, Lücker et al. [2–6], a political and scientific debate has emerged about whether the slaughter of pregnant animals can be reconciled with the requirements for a humane killing of animals. One of the major ethical concerns in this context is the perceptual awareness and viability of foetuses during the slaughter of the mother. Marahrens and Schwarzlose of Germany’s Federal Research Institute for Animal Health promote the opinion that foetuses in the 3rd trimester will have a relevant reduced welfare during the slaughter of their mother [7]. The authors state, that more research is needed to assess the observed changes in physiological, electrophysiological, and endocrinological parameters of the foetuses that are exposed to such a treatment.

In March 2014 public interest was attracted by a television report that addressed the suffering of foetuses during slaughter [8]. The increased media interest on animal welfare issues related to slaughter in Germany and abroad has caused German politicians to focus on these topics. The on-going dialogue led to a request in the course of the Standing Committee on the Food Chain and Animal Health meeting in Brussels, April 8th 2014 [9]. In response to this, a mandate was given to the European Food Safety Administration (EFSA) by the European Commission in order to investigate the scope of this problem and develop possible solutions. Additionally, if needed, the council regulation (EC) No 1099/2009 on the protection of animals at the time of slaughter should be reviewed with respect to protection of the unborn life [9].

### Appearance of the problem

The slaughter of pregnant cattle was considered negligible by the Scientific Committee on Veterinary Measures relating to Public Health in 1999 (SCVPH, [10, 11]). According to the SCVPH “meat consumption from pregnant heifers is exceptional as usually these animals are not slaughtered” [11]. Some studies, however, have since contradicted these assumptions and demonstrated that the actual numbers are much higher. Table 1 gives an overview of study results for the prevalence of slaughter of gravid cattle in Germany and some other countries [3–7, 9, 12–16]. Prevalence in German abattoirs ranges from 0.2 % ([7] referring to results of the National Association of Meat Hygiene, Animal Welfare and Consumer Protection) up to 15 % [3, 4]. In Luxembourg, Belgium and Italy the proportion of pregnant animals is 5.3 %, 10.1 %, and 4.5 % respectively [5]. In two British abattoirs, Singleton and Dobson observed that 23.5 % of the cows were pregnant [15]. As depicted in Table 1, most of the cows were in the 2nd and 3rd trimester at the time of slaughter (e.g. [13]). The fact, that hardly cows in the 1st trimester were observed may be attributed to the circumstance that early pregnancies may be overlooked on both farm and abattoir level. Generally, the data from the different studies should be interpreted with care, as different study designs may impede their comparability.

**Table 1:** Prevalence of slaughtering gravid cows in Germany and other countries – results of different authors

Number	Prevalence of slaughter gravid cows	Stage of gestation	Reference
Germany			
1	Up to 10.8 %, mean 4.3 % of cows and heifers	NS	Lücker et al. [6]
2	4.9 %	Mostly in 5th month; 38 % and 62 % in 2nd and 3rd trimester, respectively	Di Nicolo [5]
3	Up to 15 %, mean 9.6 %, median 7.1 % of cows and heifers	90 % in 2nd or 3rd trimester	Riehn et al. [3, 4]
4	0.2 % and 1.2 %	NS	Marahrens and Schwarzlose [7] referring to results of the National Association of Meat Hygiene, Animal Welfare and Consumer Protection
5	3.5 %	56 % in 2nd or 3rd trimester, 0.8 % in the 3rd trimester	German Government [9] referring the German Association of the Meat Industry
Other countries			
6	approximately 5 % (USA)	NS	Kushinsky [12]
7	NS	13.1 % in 1st, 62.6 % in 2nd and 24.3 % in 3rd trimester (Canada)	Herenda [13]
8	8.6 % (Pakistan)	NS	Khan and Khan [14]
9	23.5 % (Great Britain)	26.9 % in 3rd trimester (Great Britain)	Singleton and Dobson [15]
10	5.3 % (Luxembourg), 10.1 % (Belgium), 4.5 % (Italy)	In 3rd trimester: 36 % (Luxembourg), 15 % (Italy)	Di Nicolo [5]
11	1.5–2.1 % (Nigeria)	NS	Ademola [16]

Different authors described the prevalence of slaughtering gravid cows and their stage of gravidity in Germany and some other countries. (NS: not specified)

However, the problem cannot be underestimated in its significance, since not only cattle but also other species are effected as demonstrated by Fayemi and Muchenje [17].

### Legal and ethical aspects

There are no regulations, neither in National nor in Community law, prohibiting the slaughter of pregnant animals and governing the fate of the foetuses. Due to a supposed underestimation of the total prevalence by the SCVPH and the lack of reliable data legislators at European level have not issued any mandatory regulations on how to handle the problem [3, 4]. In addition, no options are granted to the Member States to adopt any regulations with regard to this issue at national level. Currently the only statute protecting females around the time of calving is the EU Transport regulation – Regulation (EC) 1/2005 Annex I, Chapter I Nr. 2 c [18]. It prohibits the transport of “pregnant females for whom 90 % or more of the expected gestation period has already passed, or females who have given birth in the previous week”. Riehn et al. [3, 4] indicate that determining the correct percentage of gestation is almost impossible on abattoir level, where only limited examination opportunities are available during *pre-mortem* inspection. The authors also state, that the Regulation (EC) 854/2004 [19] obliges the official veterinarian nevertheless to do a *pre-mortem* inspection (Annex I, Section I, Chapter II, B, Nr. 2 a) “to verify compliance with relevant Community and national rules on animal welfare, such as rules concerning the protection of animals at the time of slaughter and during transport” (Annex I, Section I, Chapter II, C).

The fate of foetuses is also not specifically mentioned in any regulation. However, Chapter II, Article 3 of the Regulation (EC) 1099/2009 [20] states that “animals shall be spared any avoidable pain, distress or suffering during their killing and related operations“(1) and that “business operators shall, in particular, take the necessary measures to ensure that animals (a) are provided with physical comfort and protection” and (d) “do not show signs of avoidable pain or fear or exhibit abnormal behaviour“(2a, d).

To date there is a dispute if and from which developmental stage on foetuses are conscious and sensitive to stress. Former studies, especially by Mellor et al. (e.g. [21, 22]) assume that foetuses lack such abilities. This opinion may be reviewed in the light of new scientific evidence. Bellieni and Buonocore [23] report that foetuses are able to feel stress and pain from the second half of the gestation on. The Experimental Animals Directive 2010/63/EU revising Directive 86/609/EEC on the protection of animals used for scientific purposes [24] already considers these new research results and states in recital 9 that “(…) there is scientific evidence showing that such [foetal forms of mammals] in the last third of the period of their development are at an increased risk of experiencing pain, suffering and distress, (…).” Altogether, it cannot be ruled out that foetuses feel pain, distress and other forms of suffering and therefore the slaughter of pregnant cattle in advanced gestational stages should be considered as an animal welfare problem.

The reasons on farm level for slaughtering gravid cows can only be assumed in the absence of valid data. The Federal Association of Veterinary Officers Germany presumes the following reasons (in decreasing order of importance): Slaughter by mistake with unknown pregnancy; injured animals, which cannot be used any more; economic reasons [25]. The relevance of economic aspects in this context is stressed by many authors e.g. Riehn et al., Cordes, Münch and Richter, Tierärztliche Vereinigung für Tierschutz e.V. [3, 8, 26, 27].

### Objective

Considering the heterogeneous data and study designs of previous German studies while respecting their relevance for further political development, our objectives were to i) determine the prevalence of the slaughter of pregnant cattle in Germany semi-quantitatively and ii) to investigate the occurring gestational stages and fate of the foetuses.

## Methods

### Survey design and questionnaire

For this investigation, an observational, cross-sectional study design was used. A voluntary and anonymous survey with specific questions was conducted amongst all final-year-students of the Faculty of Veterinary Medicine of the University of Leipzig from January 2010 until September 2013.

In Germany the study of veterinary medicine is regulated by the “Verordnung zur Approbation von Tierärztinnen und Tierärzten” (TAppV, [28]). The regulation stipulates that every student has to absolve a slaughterhouse-internship of at least 100 hours in three weeks under the supervision of the local competent authority. In the course of the internship, students are required to learn and practice the *ante*- and *post-mortem* inspection of cattle and pigs, taking particular care of animal welfare. Our study is therefore aimed at the observations of the veterinary trainees during their meat hygiene internship.

General information and preparation for the internship was conducted during a short obligatory briefing as part of the meat hygiene lecture. In addition to the general organizational information, students were asked to pay attention to specific aspects such as the slaughter of pregnant cattle and write a voluntary, anonymous internship report.

From January 2010 until September 2013 following their final exam in meat hygiene, all students of the faculty of veterinary medicine of the University of Leipzig were asked to participate in a survey. Participation was voluntary, anonymous, and had no influence on exam results.

**Fig. 1:**
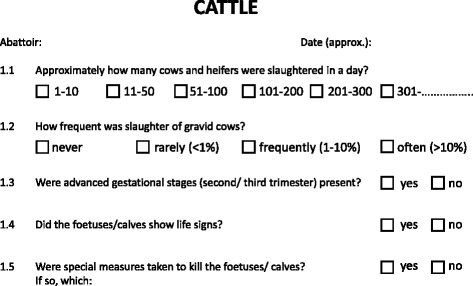
Part of the questionnaire given to the veterinary students. This translated extract of the survey shows the relevant questions for this study. It consists of 5 questions with one follow-up question

The survey consisted of two questionnaires– one for cattle and one for pigs – each containing five groups of questions about *ante-* and *post-mortem* inspection and animal welfare. For each species and abattoir the students were asked to indicate the date and abattoir of their internships. One set of questions addressed the prevalence of the slaughter of female cattle and the frequency of pregnancy amongst the cows and heifers including their stage of gestation as recognized by the students. Additionally, questions to vital signs and the fate of the foetuses were asked (see Fig. 1). Answers could be given on a nominally dichotomised or ordinal scale and for some questions free-text responses were required. The ordinal scales were based on data of previous oral reports.

### Ethical approval

Research activities involving human participants will require approval prior to their commencement. Therefore the opinion of the data protection officer of the University of Leipzig has been obtained. He confirmed an exemption from these formal requirements with regard to the following criteria: (i) The current study involves no foreseeable risk of harm or discomfort to participants and any further foreseeable risk would involve no more than inconvenience to participants, and (ii) the study involves the use of existing collections of data or records that contain only non-identifiable data about persons, entities and bodies. No names will be published.

### Data analyses

Analyses were carried out using Microsoft® Excel® 2013 (Windows)^1^ and IBM® SPSS® Statistics 22.0.^2^ The specific data of each abattoir were encrypted and abattoirs were grouped by region in order to ensure their anonymity. Only completed questionnaires were included in the initial analysis. Due to the step-by-step design of the questionnaire, the number (n) of answers for each question differs as non-responders matched the exclusion criteria and hence were dropped out from further analyses. The Somers-D-test was used to determine the association between ordinal variables. A P-value of < 0.05 was considered significant for all comparisons.

## Results

### Participants and submission (persons and the involved abattoirs)

**Fig. 2:**
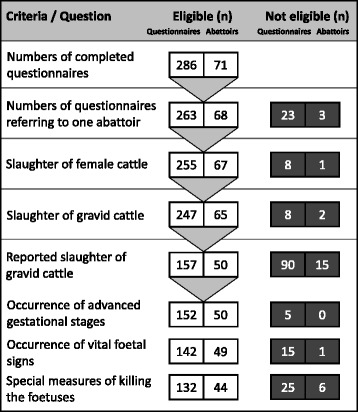
Scheme of (*n*) evaluable and non-evaluable questionnaires and encoded abattoirs of the veterinary students´ survey. Only correctly completed questionnaires are included in the analysis. Due to the design of the questionnaire the number (*n*) of answers for each question can differ as non-responders were dropped from analyses

**Fig. 3:**
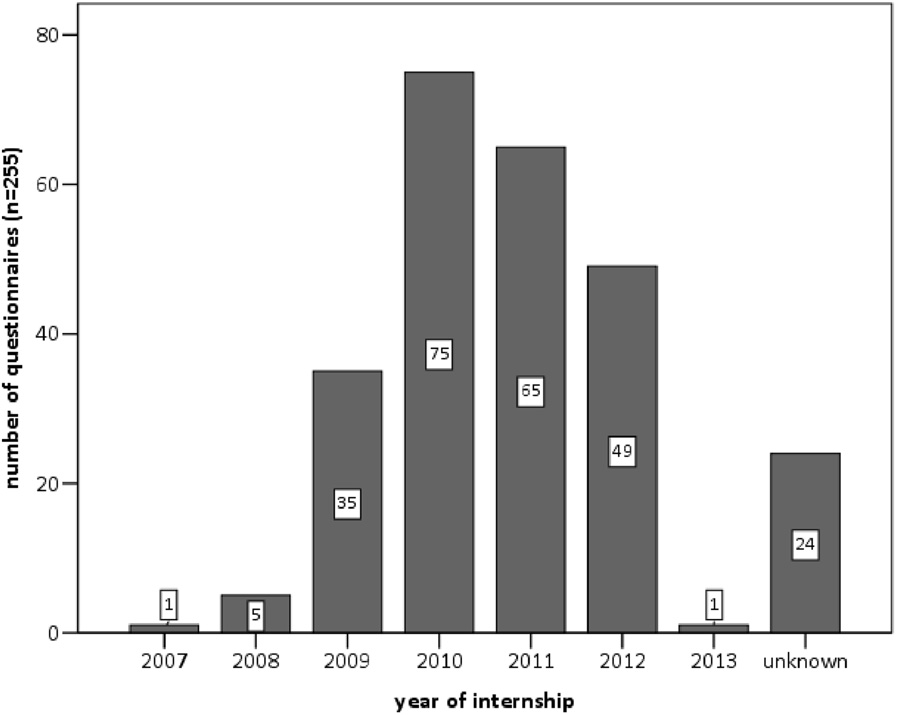
Number of valid internship questionnaires (*n* = 263) fielded in year of internship. 263 valid questionnaires have been examined. Most of the associated internships took place in 2010 (*n* = 76), 2011 (*n* = 66) and 2012 (*n* = 52). 25 dates are not specified

The survey generated 286 responses in the period from January 2010 to September 2013. All participants were students at the Faculty of Veterinary Medicine, University of Leipzig, Germany. Students were interviewed after finishing their final examination in the subject “meat hygiene”. Participation was voluntary and anonymous. Of 286 cattle-questionnaires, 263 (92 %) described the situation at one abattoir. 255 (97 %) of those 263 confirmed the slaughter of female cattle and thus fulfilled the inclusion criteria to be examined further as shown in Fig. 2. The related internships took place between 2007 and 2013. The majority however, in 2010 (n = 75; 29 %), 2011 (*n* = 65; 25 %) and 2012 (*n* = 49; 19 %), as depicted in Fig. 3. The students did their internships in 67 different slaughterhouses in Germany (*n* = 66) and abroad (*n* = 1). The 66 German abattoirs were located in 12 of the 16 federal states. The majority of the abattoirs (*n* = 39; 58 %) was located in 3 federal states; Bavaria (*n* = 14; 21 %), North Rhine-Westphalia (*n* = 14; 21 %) and Baden-Wuerttemberg (*n* = 11; 16 %).

### Numbers of slaughtered cattle and prevalence of gravid cows and heifers

The reported numbers of slaughtered female cattle are described by using a breakdown of ordinal categories. The numbers differ between the 67 abattoirs. Of the 255 eligible questionnaires, 86 (33.7 %) cases slaughtered between 1–10, 60 (23.5 %) between 11–50, 37 (14.5 %) between 51–100, 43 (16.9 %) between 101–200, 17 (6.7 %) between 201–300 and 12 (4.7 %) more than 300 female cattle per day. The majority of abattoirs are therefore small and medium-sized enterprises with a slaughter capacity of ≤50 cows/day.

In 247 (96.9 %) of the 255 questionnaires that refer to the slaughter of female cattle, the participants answered the question about the occurrence and frequency of slaughtered gravid cattle. In 157 cases (63.6 %), the slaughter of pregnant cattle was reported, although in 90 cases (36.4 %), this was never actually seen by the trainees themselves.

**Fig. 4:**
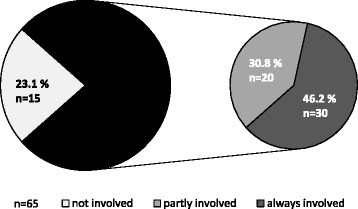
Percentage of abattoirs (*n* = 65) that are associated with slaughtering pregnant cattle. As described in the student survey, these abattoirs (*n* = 65) are never, sometimes or always associated with slaughtering pregnant cattle

**Fig. 5:**
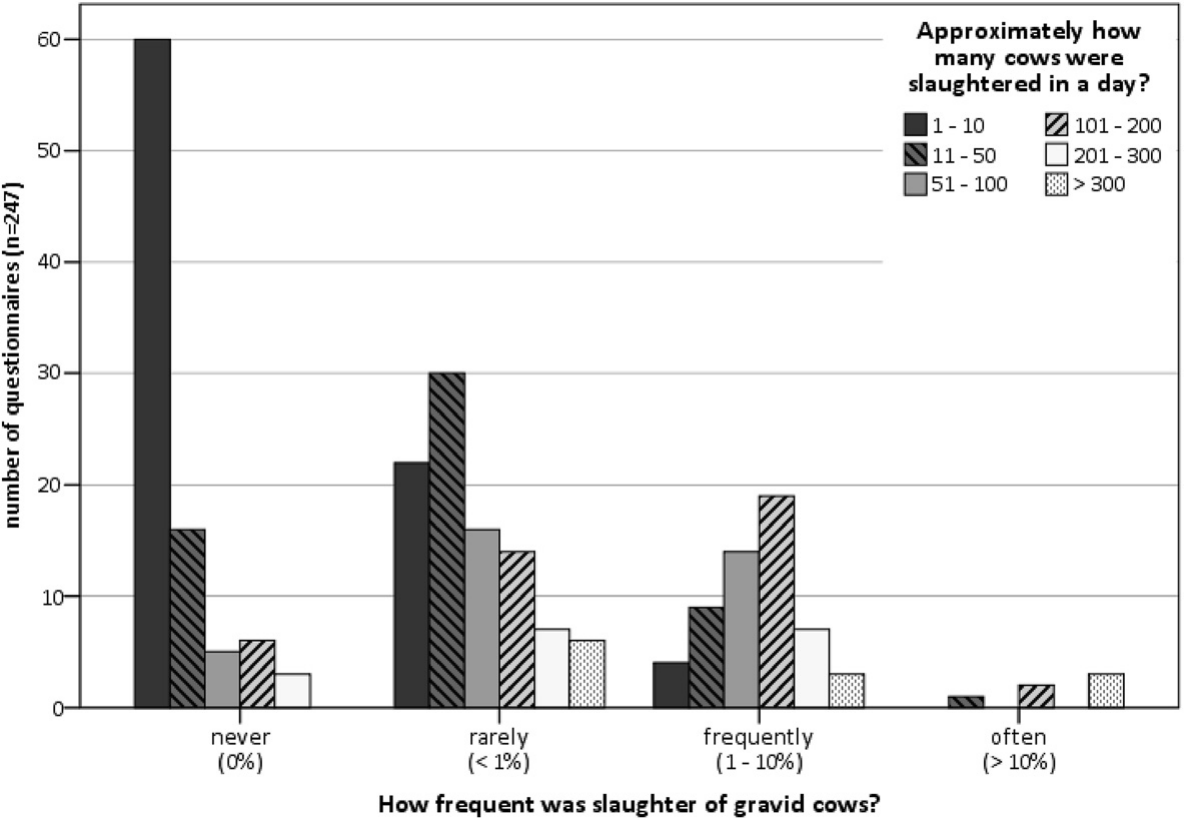
Frequency of slaughtering gravid cattle in relation to total number of female cattle slaughtered daily. This chart shows a positive tendency to slaughter gravid cows by rising number of daily slaughtered cows

An analysis of the data from the related 65 slaughterhouses shows, that there are 30 abattoirs (46.2 %) that are always reported to slaughter pregnant cattle, 20 (30.8 %) where reports vary and 15 (23.1 %) that are never reported to slaughter gravid cattle. In summary, 50 out of 65 abattoirs (76.9 %) that slaughter females also slaughter pregnant ones (see Fig. 4). However 95 of the 157 positive cases (60.5 %) reported, that the slaughter of pregnant cattle occurs rarely (<1 % of females), 56 cases (35.7 %) described it as frequently (1–10 %), and only in 6 cases (3.8 %) pregnant cows were slaughtered often (>10 %). The proportion of pregnant cattle slaughtered rises with the total number of slaughtered females (see Table 2 and Fig. 5). The correlation is significant (Somers-D 0.470, *p* < 0.01).

**Table 2:** Number of slaughtered female cattle in relation to the frequency of slaughtered pregnant cattle (*n* = 247)

		Approximately how many cows and heifers were slaughtered a day?						Sum
		1–10	11–50	51–100	101–200	201–300	>300	
How frequent was slaughter of gravid cows?	never (0 %)	60	16	5	6	3	0	90
	rarely (<1 %)	22	30	16	14	7	6	95
	frequently (1–10 %)	4	9	14	19	7	3	56
	often (>10 %)	0	1	0	2	0	3	6
Sum		86	56	35	41	17	12	247

The reported proportion (never, rarely, frequently, often) of pregnant cattle slaughtered in relation to the total number of slaughtered females per day is shown in this table

**Fig. 6:**
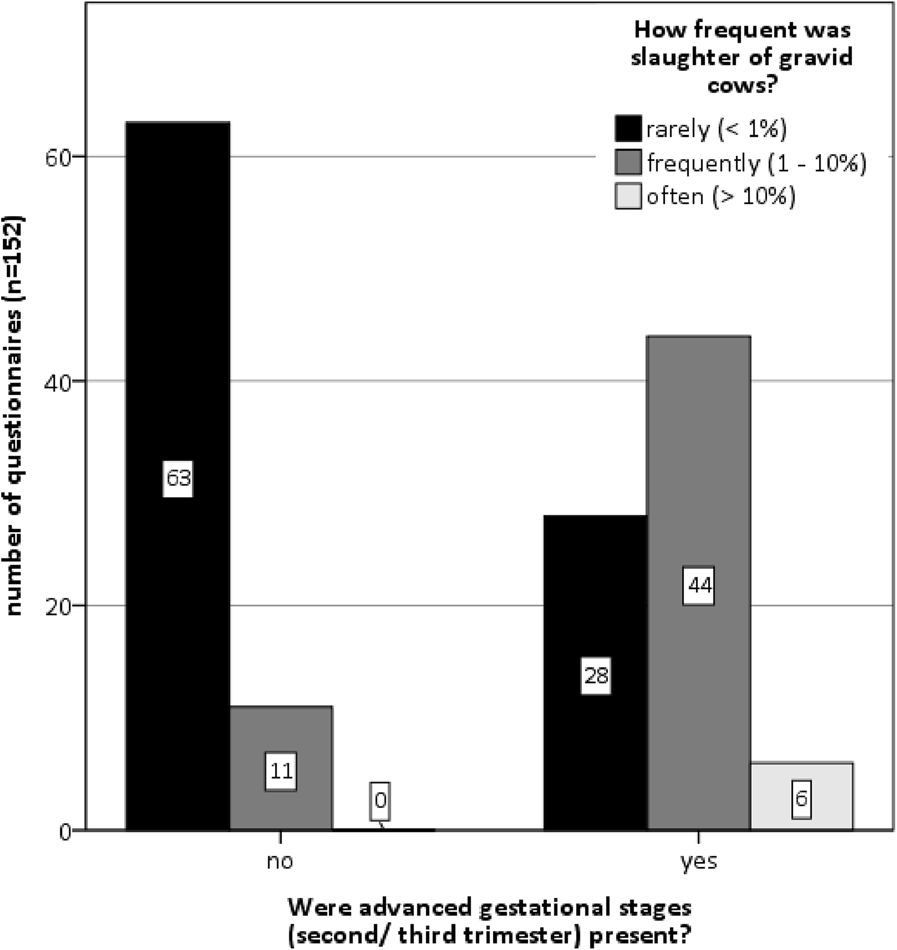
Appearance of advanced gestational stages (second/third trimester) in relation to frequency of slaughter of gravid cattle (*n* = 152). Of the 152 respondents 51.3 % reported advanced gestational stage of the slaughtered gravid cattle. This case rises significantly with the frequency of culling pregnant cattle (Somers-D 0.499, *p* < 0.01)

Enquiry was also made about the prevalence of slaughter cows in advanced gestational stages (second or third trimester). Of 152 eligible questionnaires, 78 (51.3 %) reported advanced gravidities amongst the slaughter cows. In Fig. 6, the correlation between the total number of gravid slaughter cows and the number of animals in higher gestational stages is depicted. The rarer the slaughter of pregnant cattle was, the less often advanced gravidities were reported. The correlation between those two aspects is significant (Somers-D 0.499, *p* < 0.01).

### Foetuses – vital signs and fate

Another aspect of the survey focused on the fate of the foetuses. 15 (10.6 %) of 142 respondents reported vital signs of the foetuses, such as independent movement and pulsation of the umbilical vessels. Irrespective of vital signs, one (0.8 %) of 132 respondents – as shown in Fig. 2 – reported about special measures that were taken to kill the foetus (euthanasia). The methods of euthanasia were not questioned, indeed. One student indicated that upon request, the official veterinarian stated that the foetus would die due to the cessation of uterine blood supply and that no special measures are needed. Moreover, the slaughter of pregnant farm animals also occurs to other species, as described for sheep by another student.

## Discussion

### Limitations

This survey is an observational, cross-sectional study based on students’ reports on their compulsory slaughterhouse internship, which took place 1 to 18 month before their final exam in “meat hygiene”. These time delays as well as the post exam situation are possible confounding factors that may affect the results of the study. However, all students attended a special briefing that focused on both general and specific aspects of their slaughterhouse internship. In addition, they were asked to write a voluntary, anonymous report. Therefore students were well prepared for the internship and their report served them as a helpful reminder for both, the exam preparation and the survey. In their lectures of embryology and dairy reproduction as well as in their clinical internships, the students learned to classify the stage of gravidity on the basis of the crown-rump length, eruptions of teeth, development of fur and teeth and position and size of the foetus. Thus, knowledge of these fundamentals was assumed to be compulsory.

Although the survey was conducted without knowledge of the abattoirs and the local competent authorities, this approach is acceptable. Student internship reports are common practice in many professions and the survey of students and official veterinarians on their experiences during the slaughterhouse internship is carried out since 2007. Special announcement of certain key issues would lead to bias and distortion of the data. Privacy is respected by investigation of a large number of different and anonymously evaluated abattoirs. Additionally, one should note that due to the structure of the internship (students should visit different areas of the abattoir in order to see all different aspects of meat production and inspection) the reports represent only a snapshot rather than a complete overview of the actual situation in cattle slaughtering.

### Key results and interpretation

Based on these “snapshots”, however, results show that in nearly every abattoir for cattle, females are slaughtered. Only in 8 of the 263 questionnaires (3 %), none of the given ordinal options have been selected, which could either mean that no female cattle were slaughtered or that the student did not recall or refused to answer the question. The total ratio of females amongst all cattle slaughtered is unknown and irrelevant for this survey.

With 157 of 247 eligible cases (63.6 %), the share of pregnant cattle in this study is far higher than those reported by Riehn et al., Di Nicolo, Lücker et al., Singleton and Dobson, Herenda and others [3–6, 13, 15]. Due to study design though, the data should be interpreted with care because students during their internship have limited experience regarding the assessment and interpretation of findings in comparison to an official veterinarian. In addition, these 157 questionnaires were related to only 50 abattoirs. Besides, 20 of this 50 slaughterhouses were also reported in other questionnaires, where the participant never actually saw it directly. So the information can be repeated in the same and also in other abattoirs. Moreover, a high level of attentiveness and empathy might have influenced their perception particularly with regard to health or welfare problems.

On the other hand, the aforementioned points of criticism may also be interpreted as an advantage for the present study: it offers the rare opportunity to collect information from external but yet competent observers.

Even though the semi-quantitative approach of the study does not permit an exact conclusion on the actual prevalence of the slaughter of pregnant cattle, the high number of positive reports (62 from 157; 39.5 %) shows unequivocally that the slaughter of pregnant cattle is not an exceptional event but occurs regularly even though at a different frequency of occurrence. This might also be due to the fact that large shares of the abattoirs (*n* = 146; 57.2 %) are small and medium-sized holdings with slaughter volumes of less than 50 female cattle per day. These small holdings may operate on a more local level are therefore not affected in the same way by fluctuations of suppliers on farm and transportation level as bigger, supra-regionally operating enterprises. This may result in above- or beyond-average shares of pregnant cattle in smaller plants depending of the practises in the supplying farms and the subsequent stages of the food chain. However, the present study includes many more abattoirs of different sizes and locations than former investigations on this issue.

The high proportion of questionnaires (*n* = 78; 51.3 %) reporting the slaughter of cattle during advanced gestational stages supports the findings of previous studies reporting the same phenomenon [4, 5]. However the data should be interpreted with care because differences in methodology may influence the results. An important point in this context is without doubt, that during the *ante*- and *post-mortem* inspection of slaughter cattle, later stages of gestation (late second and third trimester) are easier to diagnose then earlier stages. Furthermore, the study provides only qualitative data and is therefore not directly comparable to other studies.

A completely new aspect of this study is the focus on foetal life signs. Respondents report only in 15 of 142 cases (10.6 %) that foetal vital signs were recognizable. In only one of these cases the foetus was euthanized. This is not compatible with the animal welfare guidelines regarding the culling of pregnant animals in case of epizooty [29]. These guidelines recommend a separate euthanasia of foetuses by injection of Pentobarbital after electrical stunning of the cow. The drug diffuses through the placenta and causes a loss of consciousness and - in a high dose – paralyses the respiratory and circulatory centre leading to a rapid death [29]. Without such measures foetuses die by hypoxia. Although a number of studies [e.g. 21, 22] assume that due to low levels in foetal circulation, and the actions of other suppressors, it is unlikely that awareness occurs in the foetus during the death caused by hypoxia, a suffering of foetuses can not be completely ruled out [3, 4, 7]. It is for example questionable in this context whether only the cortex is actually involved in conscious perception or also other structures like the brainstem. In addition, is still controversial whether the prenatal measurements of EEG, which form the basis for the studies of Mellor et al. [21, 22], are comparable with the EEG reactions of an adult animal [7]. Because of these reasonable doubts the German government has tried to initiate a change of the existing European law to prevent a possible suffering of foetuses in later gestational stages [9].

## Conclusions

The results of the present study show that the slaughter of pregnant cattle is a common and widespread practice in German abattoirs. Of 255 evaluable questionnaires, 157 (63.6 %) mention the slaughter of pregnant cattle, corresponding to 76.9 % of all visited abattoirs. Slaughter of pregnant cattle is reported often (>10 %) in 6 (3.8 %), frequently (1–10 %) in 56 (35.7 %), and rarely (<1 %) in 95 (60.5 %) of all cases (*n* = 157) respectively. Hence, the SCVPH’s assumption that pregnant cattle are only slaughtered in rare exceptional cases [10, 11] can no longer be maintained. About 50 % of these animals were reported to be in the second or third stage of gestation. The high proportion of foetuses in the second and third gestational stage emphasises the relevance for animal welfare. In addition, 15 (10.6 %) of 142 questionnaires, providing information about the foetus, state that the foetus shows visible vital signs after the death of the mother. Where at present pain, suffering, and injury of the foetus during the slaughter of the mother cannot be excluded with absolute certainly, slaughter of pregnant farm animals must be considered as a pressing issue related to animal welfare. In this context suitable studies regarding (i) the prevalence of the slaughter of pregnant cattle in German and European abattoirs, (ii) the state of gestation of these animals, and (iii) the condition of the mother and the foetus with regard to welfare related parameters have to be initiated promptly. In addition, studies, regarding the sensitiveness and perceptiveness of foetuses during late pregnancy and at the time of birth have to be performed in order to elucidate the lack of scientific data in this context.

## Abbreviations

SCVPH, Scientific Committee on Veterinary Measures relating to Public Health

## Footnotes

1 Microsoft Corporation, One Microsoft Way, Redmond, WA 98052–6399, USA <https://products.office.com/de-de/home>

2 IBM Deutschland GmbH, IBM-Allee 1, 71139 Ehningen, Germany <http://www.ibm.com/de/de/>
